# Clinical Characteristics and Surgical Safety in Congenital Cataract Eyes with Three Pathological Types of Posterior Capsule Abnormalities

**DOI:** 10.1155/2020/6958051

**Published:** 2020-03-16

**Authors:** Xixia Ding, Linfeng Xiang, Qianwei Wang, Dandan Wang, Pingjun Chang, Zhangliang Li, Yinying Zhao, Feixue Chu, Chao Ma, Yun-e Zhao

**Affiliations:** ^1^Eye Hospital of Wenzhou Medical University, Wenzhou, Zhejiang, China; ^2^Key Laboratory of Vision Science, Ministry of Health P. R. China, Wenzhou, Zhejiang, China

## Abstract

**Purpose:**

To observe the clinical characteristics of 3 pathological types of posterior capsule abnormalities (PCAs) in congenital cataracts (CCs) and evaluate the surgical safety in these eyes.

**Methods:**

This study involved 239 children (367 eyes) with CC who underwent cataract surgery at the Eye Hospital of Wenzhou Medical University. All surgery videos were collected for detailed reviews. Intraoperative and postoperative complications (within 3 months) were all recorded.

**Results:**

The 3 pathological types of PCAs, namely, persistent fetal vasculature (PFV), posterior capsule defect (PCD), and posterior lenticonus (PLC), presented in 129 (35.1%) CC eyes, while 238 (64.9%) eyes were recorded as CC without PCA. The percentages of PFV, PCD, and PLC were 10.9%, 26.7%, and 5.4% in CC eyes (*n* = 367), respectively. The most common concomitant of PFV eyes was PCD (42.5%), and PFV was the most frequent (17.3%) one in PCD eyes. PLC was only associated with PFV (15%) and PCD (50%). The occurrence rates of surgical complications ranged from 0 to 5.4%, and no statistical difference was found between the eyes with and without PCA (all *P* > 0.05).

**Conclusions:**

PFV, PCD, and PLC play a very important role in the CCs. The effect of fetal vessels in PFV eyes might be an abnormally strong attachment on the posterior capsule, leading to PLC and PCD. Even in PCA patients, severe surgical complication can also be avoided with well-designed and skilled operation. This trial is registered with NCT03905044 at http://ClinicalTrials.gov.

## 1. Introduction

The management of congenital cataract (CC) is still a great challenge, and its postoperative complications such as aphakic glaucoma and secondary lens opacification still occur frequently despite the advances in microsurgical techniques and amblyopia management. Posterior capsule abnormality (PCA) plays an important role in CC formation, which is the direct cause of lens opacity. Moreover, posterior capsule problems will tremendously increase the surgical difficulty and risk of severe complications.

Persistent fetal vasculature (PFV), posterior capsule defect (PCD), and posterior lenticonus (PLC) are the most common abnormalities of the posterior capsule, which either cause incompletion or permeability transition of the capsule. Several studies [1–3] have mentioned the surgical complications in CC eyes with PFV, PCD, or PLC. However, the information given was so limited, and there is still large room for further study because of the great variability of CCs, especially in eyes with PCA.

Many studies have reported that PFV is one of the most common associations with unilateral CCs [4–8]. Additionally, CCs were also found to be combined with PCD [1] and PLC [9]. The persistent fetal vessels might lead to PCD and PLC due to the abnormally strong site between the posterior capsule and anterior hyaloid face [10]. Besides, it was also reported that PCD might begin with PLC in many patients [1].

However, these studies observed PFV, PCD, and PLC cases isolatedly. There was no study that combined PFV, PCD, and PLC cases and considered them as PCAs to figure out their relationships. The present study aims to observe the 3 different pathological types of PCAs in congenital cataracts through surgical videos and learn about their clinical characteristics and corresponding surgical complication risks. Moreover, we also tried to find out some relevance among them, and some successful surgical experience would be shared with our readers.

## 2. Methods

### 2.1. Subjects

A total of 239 children (367 eyes) with CC who underwent cataract surgery at the Eye Hospital of Wenzhou Medical University (Hangzhou, China) from 2015 to 2018 were included in the present study. All medical records were reviewed for primary diagnosis. All operations were performed by one experienced surgeon (Z. Y. E), and videos were collected for detailed reviews. Surgical complications including intra- and postoperative ones were all recorded. In the present study, the clinical data were the data within 3 months after the surgery.

The research protocol was in accordance with the Declaration of Helsinki and was approved by the Office of Research Ethics, Eye Hospital of Wenzhou Medical University.

### 2.2. Surgical Planning

All operations were performed by the same surgeon (Z. Y. E) with all the children under general anesthesia.

All the CC eyes with PCA received the lensectomy and posterior capsulotomy combined with limited anterior vitrectomy. In the eyes with PFV, in order to prevent hemorrhage, the perfusion bottle was elevated about 10–20 cm to maintain a slightly higher intraocular pressure during vitrectomy, without intraocular electric diathermy.

The surgical planning for the CC eyes without PCA was as follows: the lensectomy and posterior capsulotomy combined with limited anterior vitrectomy were performed in the younger children (<3 years); the older ones (3–6 years) received cataract phacoaspiration and posterior capsulorhexis and limited anterior vitrectomy; and for the children older than 6 years who could cooperate to receive Nd : YAG laser capsulotomy, they just received cataract phacoaspiration and the posterior capsule and vitreous were left intact.

In this study, the lensectomy, capsulotomy, and vitrectomy were all operated with the 23-gauge (23 G, 0.6 mm) vitrector through 2 corneal incisions (10 o'clock and 2 o'clock). If the primary IOL implantation was planned, a sclera tunnel incision was created at 12 o'clock.

**Figure 1:**
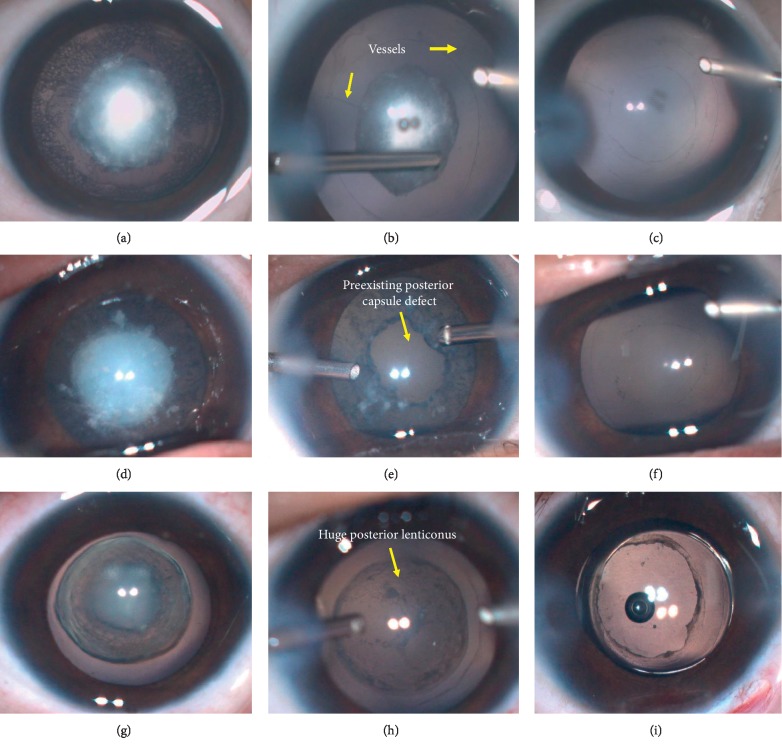
Main operative steps for 3 types of posterior capsule abnormalities (PCAs) in congenital cataract (CC) eyes. (a–c) are for persistent fetal vasculature (PFV). (a) A preoperative view; (b) after the anterior capsulotomy and major cortex removing with vitrector, the fetal vessels (yellow arrows) and the central dense plaque opacity were present clearly; (c) it was left aphakia after lensectomy combined with posterior capsulotomy and anterior vitrectomy. (d–f) are for posterior capsule defect (PCD). (d) A preoperative view; (e) a large and irregular preexisting PCD (yellow arrows) was shown after anterior capsulotomy and central cortex removed with vitrector; (f) the irregular PCD was trimmed to be a round one and followed by the anterior vitrectomy without primary intraocular lens (IOL) implantation. (g–i) are for posterior lenticonus (PLC). (g) A preoperative view; (h) the huge PLC (yellow arrows) turned up after the anterior capsulotomy and cortex removed with vitrector; (i) a single-piece IOL was implanted in the capsule bag which was followed by the capsulotomy of PLC and anterior vitrectomy.

Figure 1 shows several simple steps of the surgery (details in Videos [Supplementary-material supplementary-material-1]–[Supplementary-material supplementary-material-1]).

### 2.3. Video Reviews

**Figure 2:**
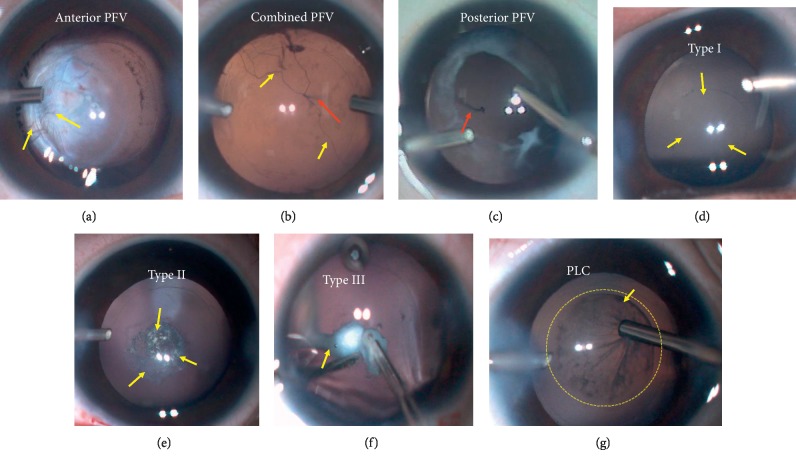
(a–c) Intraoperative views for 3 types of persistent fetal vasculature (PFV), including anterior (a), combined (b), and posterior (c) PFVs. Fetal vessels were visible on the photos. Yellow arrows point out the fetal vessels from anterior segments, while red arrows point out the vessels from the posterior segments. (d–f) Intraoperative appearances for 3 types of posterior capsule defect (PCD). Type I: a large defect with sinking cortex in the anterior vitreous; type II: a cluster of fine defects in the posterior capsule; type III: a defect with a concurrent persistent fetal vasculature (PFV). (g) Intraoperative sight for posterior lenticonus (PLC). The yellow arrow shows the involved areas of PLC.

Based on the preoperative examinations, some cases of PCA in CC eyes could be primarily diagnosed before the surgery. However, most cases were confirmed during the surgery. The diagnosis of PFV was based on the presence of 1 or more of the following clinical features: one or more persistent fetal vessels spreading on the posterior capsule, an elevated vitreous membrane or stalk from the optic nerve, retrolental fibrovascular membrane, or elongated ciliary processes. PFV is usually subdivided into anterior, posterior, or combined subtypes (Figure 2) [11].

The diagnosis of PCD was confirmed intraoperatively in the light of a preexisting defect in the posterior capsule, during or after common aspiration of the lens cortex [12]. In the current study, we noted three types of PCD (Figure 2), as stated in our previous study [12]: type I: a large defect with sinking cortex in the anterior vitreous; type II: a cluster of fine defects in the posterior capsule; and type III: a defect with concurrent PFV.

All PLC cases were defined as having a posterior capsule bulge (Figure 2).

### 2.4. Statistical Analysis

Statistical analysis was performed using the Windows version of SPSS 19.0 (SPSS Inc., Chicago, IL, US). The Kolmogorov–Smirnov test was used to check the normal distribution of variables. Values were expressed as mean ± SD or median with ranges (nonnormal distributions). The occurrence rates of complications were compared using the chi-squared tests. *P* values less than 0.05 were considered statistically significant.

## 3. Results

This study included 367 eyes from 239 children with CC, and there were 136 (56.9%) boys and 103 (43.1%) girls. The median surgical age was 10 months (range: 1–181 months). Unilateral cataract was found in 111 children (111/239, 46.4%) and 128 children (128/239, 53.6%) suffering from bilateral cataracts.

Concomitant systemic abnormalities were also found in the present study, including anal atresia, severe jaundice, cytomegaloviral pneumonitis, nervous deafness, Down's syndrome, amentia, severe malnutrition, cheilopalatognathus, palatoschisis, laryngeal cartilage hypoplasia, and congenital heart disease. Each of the aforementioned abnormalities occurred only in one patient (1/239, 0.42%), except for laryngeal cartilage hypoplasia (2/239, 0.84%). In total, 12 patients (12/239, 5.0%) were diagnosed with concomitant systemic abnormalities.

The PCAs (PFV + PCD + PLC) presented in 129 (35.1%) CC eyes, while 238 (64.9%) eyes were recorded as CCs without any PCA. The percentages of PFV, PCD, and PLC were 10.9%, 26.7%, and 5.4% in CC eyes (*n* = 367), respectively (Table 1).

**Figure 3:**
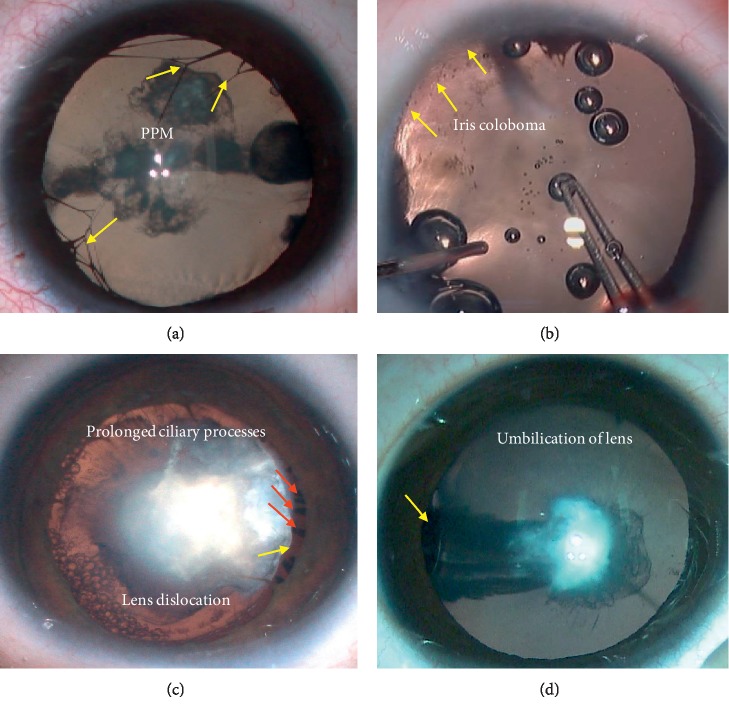
Some ocular concomitant of persistent fetal vasculature (PFV) in congenital cataract (CC) eyes: (a) yellow arrows show persistent pupillary membrane (PPM); (b) iris coloboma (yellow arrows); (c) the prolonged ciliary processes (red arrows) are visible, and the exposed equator of lens is pointed out by the yellow arrow (lens dislocation); (d) the yellow arrow shows the umbilication of lens.

**Figure 4:**
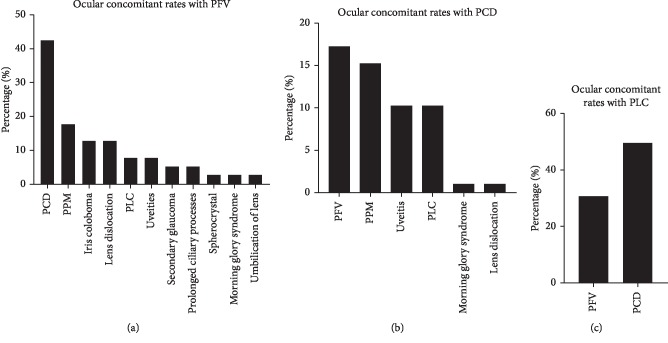
The ocular concomitant rates in congenital cataract (CC) eyes with posterior capsule abnormality (PCA), including persistent fetal vasculature (PFV), posterior capsule defect (PCD), and posterior lenticonus (PLC): (a) PFV is often associated with PCD, persistent pupillary membrane (PPM), iris coloboma, lens dislocation, PLC, uveitis, secondary glaucoma, prolonged ciliary processes, spherocrystal, morning glory syndrome, and umbilication of lens; (b) PCD is often combined with PFV, PPM, uveitis, PLC, morning glory syndrome, and lens dislocation; (c) PLC concomitant is PFV or PCD.

Table 2 shows that, in the CCs combined with PFV, there were 3 different types including anterior (27.5%), posterior (32.5%), and combined (40%) PFV. Many CC eyes with PFV also showed other ocular malformations including PCD, PLC, persistent pupillary membrane (PPM), uveitis, iris coloboma, and lens dislocation (Figures 3 and 4). The most common concomitant of PFV was PCD (17/40, 42.5%), followed by PPM (7/40, 17.5%), as shown in Figure 4.

Table 3 shows that type I of PCD was the most common, while type III; (PFV) was the least. A lot of PCD eyes were associated with other ocular malformations including PFV, PPM, uveitis, PLC, morning glory syndrome, and lens dislocation (Figure 4). Among these malformations, PFV was the most frequent (17/98, 17.3%), followed by PPM (15/98, 15.3%).

Figure 4 also shows that PLC was only associated with PFV (3/20, 15%) and PCD (10/20, 50%).

**Figure 5:**
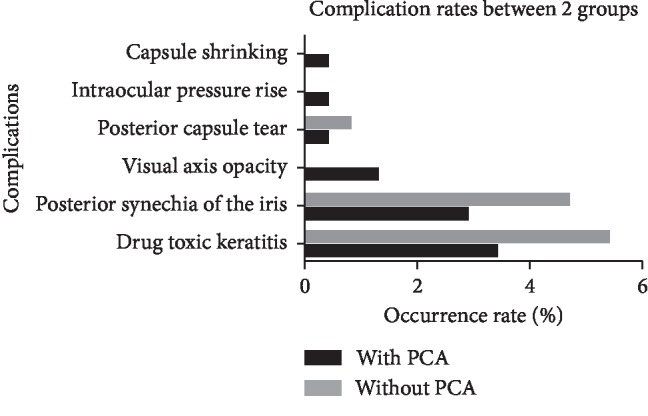
The comparison of surgical complication rates between the eyes with and without posterior capsule abnormality (PCA).

In this study, a few surgical complications were observed, including posterior capsule tear, posterior synechia of the iris, visual axis opacity (VAO), intraocular pressure (IOP) rise (it occurred only in one eye, and the reason might be caused by the adverse drug reaction of topical application of dexamethasone. The IOP went back to the normal level gradually after the dose reduction of dexamethasone), drug toxic keratitis, and capsule shrinking. The occurrence rates of all different complications were shown between eyes with and without PCA in detail in Table 4 and Figure 5, respectively. All the rates were very low which ranged from 0 to 5.4%, and there was no statistical difference of the rates between the eyes with and without PCA (all *P* > 0.05).

## 4. Discussion

Surgical managements of CC are complicated and challenging, especially in the cases of eyes with PCAs, including PFV, PCD, and PLC. It will be of great importance to decide surgical plans and to avoid some surgical complications. In addition, the exact reasons for these congenital abnormalities remain unknown. The present study aims to observe the clinical characteristics of PCAs in CCs and evaluate the surgical safety in CC eyes with PCA. Then, we would figure out the relevance between PFV, PCD, and PLC and also provide some useful surgical treatment techniques for clinicians.

In this study, the PCA (PFV, PCD, and PLC) occurrence rate was found to be as high as 35.1% in CCs during surgery, which has never been reported in the current literature. PCD showed the highest frequency of 26.7% in CC eyes. PFV was the second (10.9%), and PLC was the least (5.4%). Similar to our results, it was reported in prior studies that the incidence of PFV in unilateral cataracts was 11% to 30%, [4–8, 13–16], while bilateral PFV is uncommon with its incidence of 2.4% to 16.7% [14, 17–21]. In line with the literature [11], we also found the combined PFV was much more frequent than the anterior or posterior PFV.

However, another study [1] evaluated 400 eyes consecutively and found 27 eyes was confirmed with preexisting PCD (27/400, 6.8%), which was much lower than our results (26.7%). The reason might be that this study involved three types of PCDs according to our previous study of PCD [12]. Our surgical findings also revealed that the potential cause of the plaque opacity on the posterior capsule might be cribriform PCD (type II) or PFV. Additionally, some membranous cataracts might begin with PCD, and then the cortex would be absorbed. Three types of PCDs were firstly introduced in our former study [12]. Of 42 PCD eyes from 30 CC patients (≤1 years old), the prevalence of the 3 types was as follows: type I (large defect with sinking cortex in anterior vitreous, 38.1%), type II (cluster of fibrotic spots in posterior capsule, 47.6%), and type III (with concurrent PFV, 14.3%). However, in this study, which involved much more samples of 98 PCD eyes, we found that the rates of the aforesaid 3 types were 48.0%, 33.7%, and 18.4%, respectively. In addition, it was reported in a study [3] of 415 nontraumatic cataract cases with primary IOL implantation that the percentage of PLC was 7.7% (32/415), which was similar to the result of this study (5.4%). The posterior capsule thinning and bending in cases of PLC will result in progressive distortion of the lens fibers and cataract formation [22, 23]. Also, there are many theories explaining the development of PLC including subepithelial capsule hyperplasia, embryologic hyaloid artery traction, [24] inherent weakness of the posterior capsule wall, and the excessive strain of accommodation, which will result in posterior herniation of the lens.

In the present study, we found that PCD was the most common concomitant (42.5%) in PFV eyes, and the most common accompanist was PFV (17.3%) in PCD eyes. In addition, PLC was only associated with PFV (15%) and PCD (50%). Therefore, we hypothesize that PFV, PCD, and PLC may have the same etiology involving the abnormal dissolving of fetal vessels. PFV occurs when the fetal vessels fail to dissolve. If these vessels have gone through the posterior capsule, PCD is present. Then, the inflammatory reaction mechanism is stimulated, which is followed by the plaque fibrosis membrane. Central posterior dense lens opacity may provide a clue for PFV or PCD before the surgery. If the pink opacity is present, the opacity may associate with vessels which mean PFV. Furthermore, it has been reported that the posterior capsular plaques are a result of PFV with presence of the classic hyaloid remnants, which are though not commonly observed in eyes [25]. They ascribed the reason to the timing of hyaloid dissolution. Mullner-Eidenbock and colleagues [7] hypothesized that the hyaloid simply dissolves more slowly than expected and then fetal vessels perforating the lens capsule are present during lens development, thus resulting in a strong adherence of the lens to the posterior capsule. At last, the vessels subsequently resolve, but the resulting lens opacity and posterior capsular plaque remain. The persistent hyaloidal stalk is integrated anteriorly into posterior lens capsule, and the posterior traction of the lens capsule can lead to posterior lenticonus [26]. Additionally, Franceschetti and Rickli [27] found overgrowth or aberrant hypertrophy of the posterior lens cortex, and this overgrowth of lens fibers forces backward displacement of a thin, defective posterior capsule. Mistr et al. [3] found that preoperative posterior capsule rupture was noted in 8/32 posterior lentiglobus cases (25%). Furthermore, Vasavada et al. [1] also observed a higher incidence of unilateral preexisting PCDs, which perhaps begin as posterior lenticonus.

In our study of 3-month postoperative follow-up, no anterior or vitreous hemorrhage was found in any eyes and no severe complication, such as retinal detachment, happened which might cause fatal visual loss even with timely and effective treatments. Obviously, the risk of terrible complications also depended on the severity of PFV. Kuhli-Hattenbach et al. [2] reviewed 19 CC eyes with PFV and recorded the surgical complications including vitreous hemorrhage (52.6%), retinal detachment (15.8%), aphakic glaucoma (10.5%), secondary cataract (10.5%), fibrinous reaction (10.5%), and anterior chamber hemorrhage (5.2%). Compared to PFV, the surgical complications of PCD and PLC were reported relatively slight, including posterior synechiae (3.70%), peripheral anterior synechiae (3.70%), intraoperative posterior capsule tear (3.13%), posterior synechiae (3.13%), VAO (3.13%), and abrupt postoperative increase in IOP (3.13%) [1, 3].

In the present study, the incidence of surgical complications was very low even in the CC eyes with PCAs and it should be contributed to the well-designed surgical plans and highly skilled operative techniques. As we all know, posterior capsulotomy and partial anterior vitrectomy have been widely accepted for avoiding future opacification of visual axis especially in children younger than 6 years [28]. However, both the posterior capsule and anterior vitreous of eyes with PCA also should be handled even for children older than 6 years. Thus, first of all, the surgeon must be highly skilled for both cataract surgery and anterior vitrectomy. Secondly, transcorneal 23G lensectomy was preferred instead of phacoaspiration for the small incision size, anterior chamber stability, and hand piece rigidity [29].

Sometimes, fetal vessels cannot be detected with preoperative examinations, and the PFV diagnosis should be confirmed during the operation. PFV always comes along with other ocular malformations, such as PPM, iris coloboma, and centrally dragged ciliary processes. Therefore, if these concomitants occur or white plaque mixed with pink mass is found during the surgery, it is very likely to be the symptom of PFV. We used lensectomy and anterior vitrectomy to effectively avoid the traction to posterior capsule plaque and vessels. Then, more attention should be paid to the intraocular bleeding. Usually, we raised perfusion pressure for hemostasis through elevating IOP when cutting the fetal vessel membrane. Besides, electrocoagulation hemostasis should be taken into consideration in some severe and complicated cases. Moreover, all the incisions were sutured to prevent the intraocular bleeding resulted from postoperative hypotony.

It has been reported that the signs of preexisting PCD were the presence of demarcated, thick defect margins and white dots on the posterior capsule and in the anterior vitreous (fish-tail sign) [1]. Additionally, we have also found that a decrease in lens thickness was an independent predictor of preexisting PCD in CC [12]. If some of the above characteristics are observed, lensectomy is recommended instead of phacoaspiration. Moreover, the procedure should be conducted in a gentle way to avoid enlarging the preexisting PCD and reducing the cortex drop. The PCD with sharp corners was very likely to tear. Thus, all the sharp corners should be trimmed into a circle to resist the rupture. Besides, the cortex falling into the vitreous could be removed efficiently and completely using the vitrector without posing much traction to the margin of PCD and vitreous. Then, the area of PCD was not likely to be enlarged, and this was very important to the in-bag IOL stability. When the size of PCD was too large, IOL sulcus implantation with optic capture or in-bag implantation with reserve optic reverse capture by anterior capsulorhexis could be made to lock the IOL.

For the CC eyes with PLC, the procedure also should be conducted gently for the posterior cone was very weak and was easy to rupture. In fact, it always happens with PCD, and the surgery is increasingly challenging. The lensectomy and anterior vitrectomy could also reduce the posterior capsule disturbance, and posterior vitrectorhexis could better control the opening size.

To sum up, according to the points mentioned above, it was vital to obtain some clues through careful examination before operation and to make preoperative diagnosis as far as possible. Then, the surgical planning could be made, and the procedure could be fully prepared before the surgery. Consequently, the complications would be greatly reduced.

This study also has several limitations. Firstly, the patient sample of only one surgeon is limited, and a multicentral clinical study is necessary for further study. Secondly, only the postoperative data of the first 3 months were involved in the present study, and long-term surgical safety should be evaluated in the next study. Thirdly, the clinical outcomes of visual rehabilitation should be analyzed as well in the future.

## 5. Conclusions

PCAs, PFV, PCD, and PLC play a very important role in the CC in pediatric patients. It has been always noted that the CC is associated with one or more abnormalities. The effect of fetal vessels in PFV eyes might be that the vessels act as an abnormally strong attachment on the posterior capsule, forming PLC, PCD, or even lens opacities directly. Additionally, PCDs might begin with PLCs and even no posterior lenticonus area can be found in PCD eyes. Even in these complicated eyes, severe surgical complication can also be avoided through proper surgical treatment.

## Figures and Tables

**Table 1:** Congenital cataract with posterior capsule abnormalities including PFV, PCD, and PLC.

Congenital cataract	Number of eyes (*n* = 367)	Percentage
PFV	40	10.9
PCD	98	26.7
PLC	20	5.4
Total (PFV + PCD + PLC)	129	35.1

PFV, persistent fetal vasculature; PCD, posterior capsule defect; PLC, posterior lenticonus.

**Table 2:** PFV types.

	Number of eyes (*n* = 40)	Percentage
Type		
Anterior	11	27.5
Posterior	13	32.5
Combined	16	40.0

PFV, persistent fetal vasculature.

**Table 3:** PCD types.

	Number of eyes (*n* = 98)	Percentage
Type		
I	47	48.0
II	33	33.7
III	18	18.4

Type I: a large defect with sinking cortex in the anterior vitreous; type II: a cluster of fine defects in the posterior capsule; type III: a defect with a concurrent persistent fetal vasculature. PCD, posterior capsule defect.

**Table 4:** The comparison of surgical complication rates between the eyes with and without PCA.

	With PCA (*n*, %)	Without PCA (*n*, %)	*P* ^*∗*^
Capsule shrinking	0	0.4	—
IOP rise	0.4	0	—
Posterior capsule tear	0.4	0.8	0.659
VAO	1.3	0	—
Posterior synechia of the iris	2.9	4.7	0.397
Drug toxic keratitis	3.4	5.4	0.340

PCA, posterior capsule abnormality; IOP, intraocular pressure; VAO, visual axis opacity; ^*∗*^chi-squared test.

## Data Availability

The data in this study are all from children or infants, and the access to the data is restricted for patient privacy. The readers can contact Dr. Ding (dingxixiaxc@163.com) for more details.

## Supplementary Materials

Supplementary Materials Video 1: a brief surgical procedure for a congenital cataract (CC) eye with persistent fetal vasculature (PFV, combined) and posterior capsule defect (PCD, type III). Firstly, the anterior capsulotomy was performed, and the major cortex was removed with vitrector. Then, the fetal vessels from anterior segments and the central dense plaque opacity were present clearly. A large fetal vessel from posterior segments and a significant PCD appear immediately after peeling off the dense fibrous membrane. At last, posterior capsulotomy and anterior vitrectomy were performed, and it was left aphakia. Video 2: a brief surgical procedure for a congenital cataract (CC) eye combined with posterior capsule defect (PCD, type I). A large and irregular pre-existing PCD was shown after anterior capsulotomy and central cortex being removed with vitrector. Then, the irregular PCD was trimmed to be a round one using the vitrector. At last, the anterior vitrectomy was performed without primary intraocular lens (IOL) implantation. Video 3: a brief surgical procedure for a congenital cataract (CC) eye combined with posterior lenticonus (PLC). The huge PLC turned up after the anterior capsulotomy and cortex being removed with vitrector. After that, a single-piece IOL was implanted in the capsule bag. Then, the capsulotomy of PLC and anterior vitrectomy were performed immediately.
